# Evolution of extensively drug-resistant *Mycobacterium tuberculosis* from a susceptible ancestor in a single patient

**DOI:** 10.1186/s13059-014-0490-3

**Published:** 2014-11-07

**Authors:** Vegard Eldholm, Gunnstein Norheim, Bent von der Lippe, Wibeke Kinander, Ulf R Dahle, Dominique A Caugant, Turid Mannsåker, Anne Torunn Mengshoel, Anne Ma Dyrhol-Riise, Francois Balloux

**Affiliations:** Division of Infectious Disease Control, Norwegian Institute of Public Health, Lovisenberggata 8, 0456 Oslo, Norway; Department of Infectious Diseases, Oslo University Hospital, 0450 Oslo, Norway; Institute of Clinical Medicine, Faculty of Medicine, University of Oslo, 0450 Oslo, Norway; UCL Genetics Institute, Department of Genetics, Evolution and Environment, University College London, London, WC1E 6BT UK

## Abstract

**Background:**

*Mycobacterium tuberculosis* is characterized by a low mutation rate and a lack of genetic recombination. Yet, the rise of extensively resistant strains paints a picture of a microbe with an impressive adaptive potential. Here we describe the first documented case of extensively drug-resistant tuberculosis evolved from a susceptible ancestor within a single patient.

**Results:**

Genome sequences of nine serial *M. tuberculosis* isolates from the same patient uncovered a dramatic turnover of competing lineages driven by the emergence, and subsequent fixation or loss of single nucleotide polymorphisms. For most drugs, resistance arose through independent emergence of mutations in more than one clone, of which only one ultimately prevailed as the clone carrying it expanded, displacing the other clones in the process. The vast majority of mutations identified over 3.5 years were either involved in drug resistance or hitchhiking in the genetic background of these. Additionally, RNA-sequencing of isolates grown in the absence of drug challenge revealed that the efflux-associated *iniBAC* operon was up-regulated over time, whereas down-regulated genes include those involved in mycolic acid synthesis.

**Conclusions:**

We observed both rapid acquisitions of resistance to antimicrobial compounds mediated by individual mutations as well as a gradual increase in fitness in the presence of antibiotics, likely driven by stable gene expression reprogramming. The rapid turnover of resistance mutations and hitchhiking neutral mutations has major implications for inferring tuberculosis transmission events in situations where drug resistance evolves within transmission chains.

**Electronic supplementary material:**

The online version of this article (doi:10.1186/s13059-014-0490-3) contains supplementary material, which is available to authorized users.

## Introduction

The evolution of drug resistance is a major impediment to current anti-tuberculosis efforts. Despite the low *in vitro* mutation rate of *Mycobacterium tuberculosis* [1,2], cases of extensively drug-resistant tuberculosis (XDR-TB) are now frequently reported [3,4]. Multidrug-resistant tuberculosis (MDR-TB) is defined as isolates resistant to at least the first-line drugs isoniazid (INH) and rifampicin (RIF), whereas XDR-TB requires an MDR phenotype with additional resistance to any fluoroquinolone (FLQ) and at least one of the second-line injectable drugs capreomycin (CPR), kanamycin (KAN or amikacin (AMK) [5]. By the end of 2011, 77 countries had reported at least one case of XDR-TB, and about 9% of the approximately 650,000 MDR-TB cases worldwide qualified for XDR-TB status [6]. Evidence of ongoing transmission of XDR-TB and so-called totally resistant strains [3] makes the situation all the more grave.

Emergence of drug resistance is generally attributed to poor patient compliance with the standard multi-drug regimen, yet the stepwise evolution of drug resistance despite stringent adherence to a directly observed treatment (DOT) protocol has been reported in a HIV co-infected mining community [7]; in fact, the introduction of the DOT and DOT plus programs in the absence of drug susceptibility testing (DST) might have been instrumental for the evolution of XDR-TB [8]. Adding to this, clinical trial simulations with a hollow fiber tuberculosis (TB) model demonstrated that approximately 1% of TB patients with perfect adherence would still develop MDR-TB due to pharmacokinetic variability alone [9]. Emergence of drug resistance is a stepwise process and the evolution of MDR-TB from a susceptible isolate or from MDR-TB to XDR-TB in a single patient is not entirely uncommon (for example, [10–12]).

**Figure 1:**
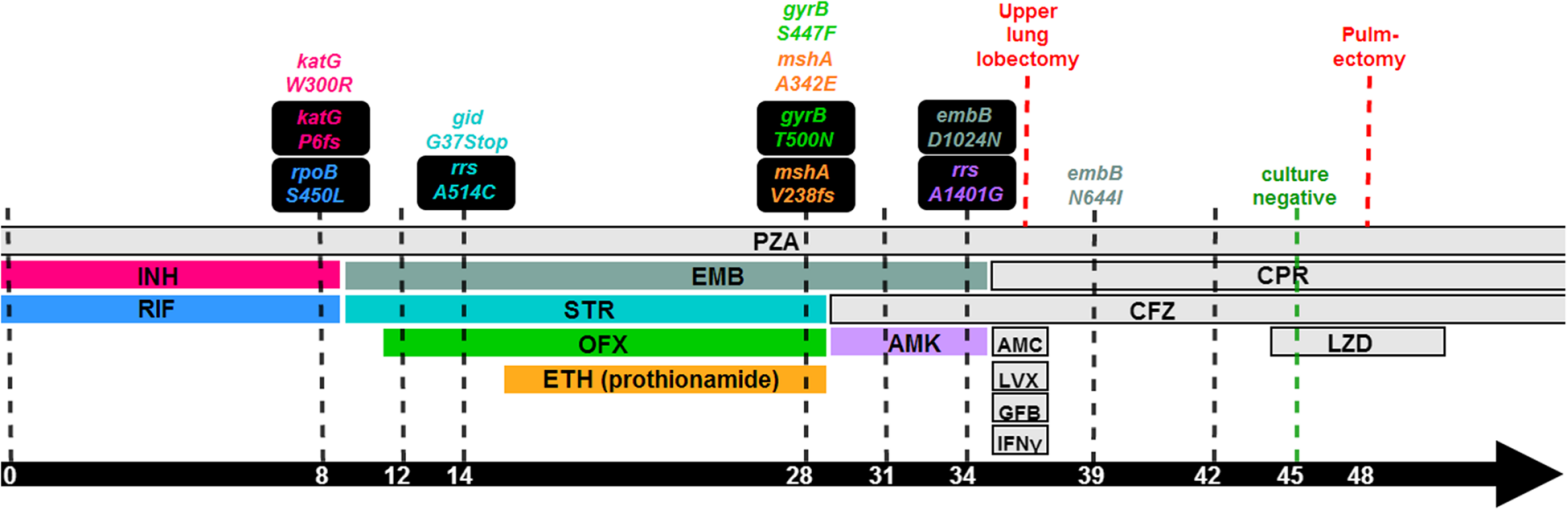
**Time-line of clinical interventions and drug resistance acquisition.** Drug regimen is indicated by horizontal bars. Black dashed lines indicate available clinical isolates. Above the dashed lines, the first instances of resistance-conferring mutations identified in the corresponding clinical isolates are indicated. The mutations are colored to match the drug to which it confers resistance. Black boxes indicate mutations that were ultimately fixed (>85% of reads in SF9), whereas non-boxed mutations indicate transient mutations. INH, isoniazid; RIF, rifampicin; PZA, pyrazinamide; EMB, ethambutol; STR, streptomycin; OFX, ofloxacin; ETH, ethionamide; CFZ, clofazimine; AMK, amikacin; AMC, amoxicillin/clavulanate; LVX, levofloxacin; GFB, gemfibrozil; IFNγ, gamma interferon; CPR, capreomycin; LZD, linezolid. Frameshift mutations are denoted as fs.

Here we report a detailed investigation of what to our knowledge is the first published case of XDR-TB evolved from a susceptible ancestor in a single patient, highlighting the impressive adaptive potential of *M. tuberculosis*. We analyzed the genomes of nine consecutive *M. tuberculosis* isolates from the same patient, recovered over a 42 month period (Figure 1), and found that resistance mutations were acquired multiple times by individual clones. The strain developed resistance to INH, RIF, streptomycin (STR), FLQ, ethionamide (ETH) and AMK as well as low-level resistance to ethambutol (EMB). Surprisingly high genetic divergence was detected between isolates collected over short time spans, reflecting the rapid expansion and collapse of different clones evolving in parallel. Finally, RNA-sequencing revealed that transcriptional regulation of drug efflux and mycolic acid synthesis may be involved in increased growth efficiency in the presence of antibiotics.

## Results and discussion

### Microevolution of *M. tuberculosis* serial isolates

The genomes of the nine clinical isolates from the same patient, representing various levels of phenotypic drug resistance, from susceptible to XDR-TB were sequenced at a median depth of 210× coverage. We applied SNP-calling parameters appropriate for the detection of multiple clones present in any given clinical isolate. Over the course of 42 months from diagnosis, we identified 35 mutations with a SNP frequency of >25% in at least one isolate (Additional file 1), of which 20 were transient and 15 went to fixation. Twenty-four mutations reached a frequency of >70% in least one isolate, which is a more typical threshold for SNP calling. Among the full set of 35 mutations, 12 could be assigned a role in conferring drug-resistance (Table 1).

**Table 1:** **Resistance mutations acquired over time in serial isolates from patient**

**Gene**	**Protein/RNA change**	**Resistance**	**Reference**
*gyrB*	S447F	FLQ	[13]
*gyrB*	**T500N**	FLQ	[14]
*mshA*	**V238 frameshift**	ETH	[15]
*mshA*	A342E	ETH	[15]
*rpoB*	**S450L**	RIF	[16]
*rrs*	**A514C**	STR	[17]
*gid*	G37 Stop	STR	[18]
*rrs*	**A1401G**	AMK/KAN	[19]
*katG*	W300R	INH	[20]
*katG*	**P7 frameshift**	INH	[21]
*embB*	N644I	EMB	
*embB*	**D1024N**	EMB	

Mutations eventually reaching fixation are highlighted in bold.

**Figure 2:**
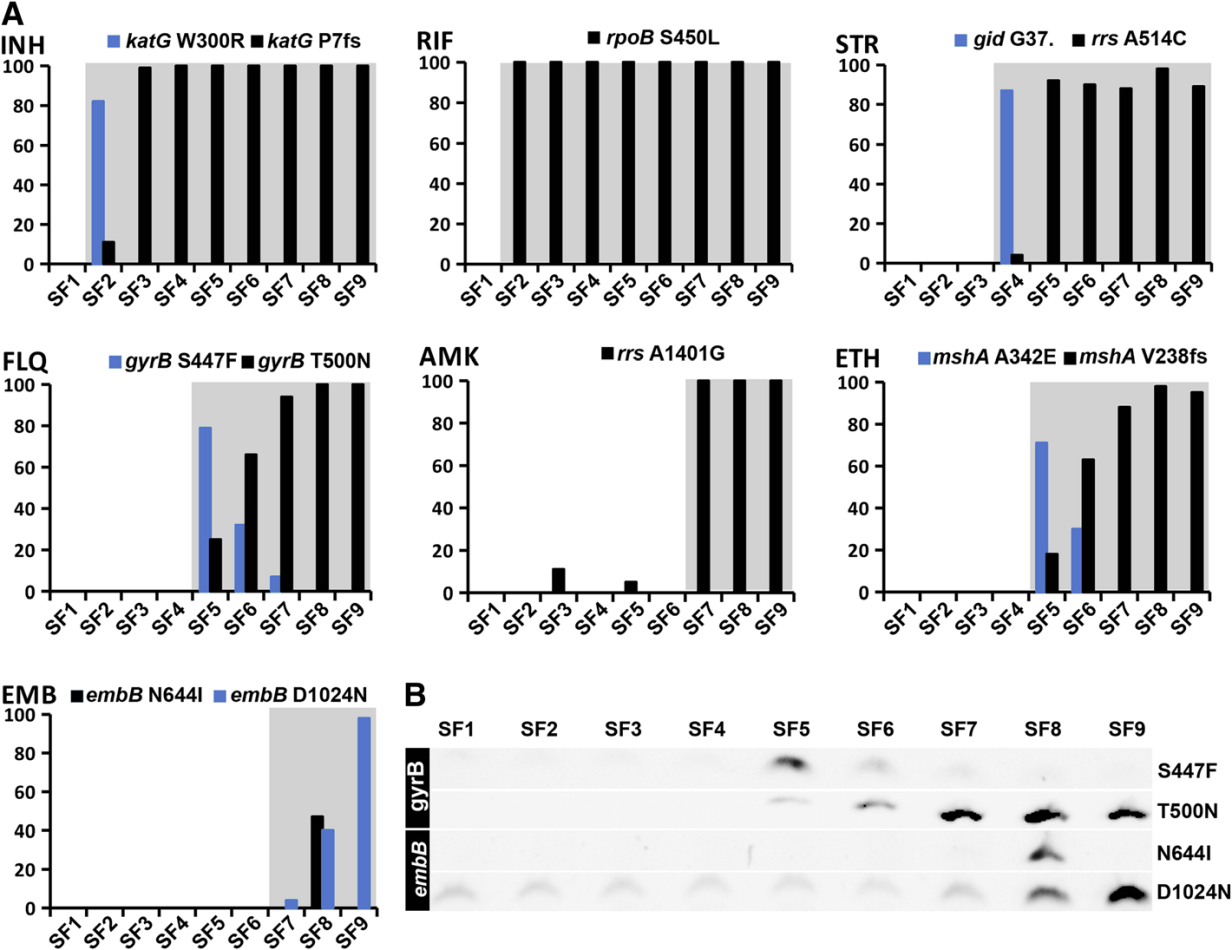
**Frequency of resistance mutations in serial isolates. (A)** Frequency of resistance mutations in sequencing reads from serial isolates. Grey background shading indicates that the isolate was resistant to the given drug on the BD BACTEC 460 platform using standard critical concentrations, with the exception of EMB where the shading indicates resistance at 2 μg/ml on the BD MGIT 960 platform (see main text for details). The FLQ resistant isolates were resistant to both ofloxacin and ciprofloxacin. **(B)** PCR verification of selected resistance mutations, separated and visualized on agarose gel.

**Figure 3:**
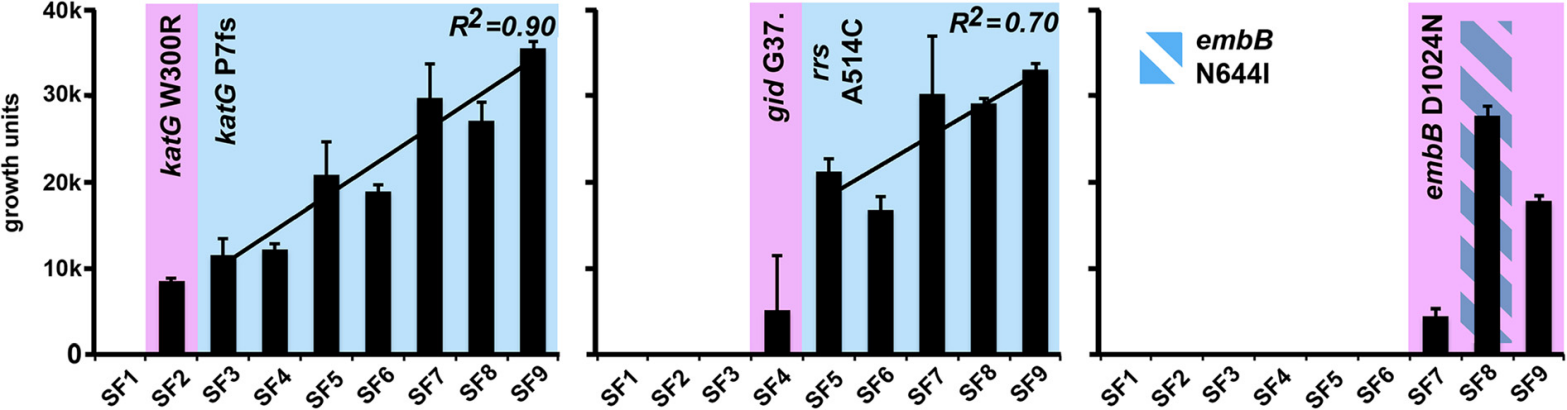
**Growth efficiency in the presence of antibiotics of clinical isolates harboring different resistance mutations.** Growth in antibiotics relative to untreated controls (from the left: INH, STR, EMB). See main text for details. Color shading indicates the specific resistance mutation present in each isolate. Isolate SF8 contains a mixture of two populations with two different *embB* mutations.

Phenotypic resistance to INH, RIF, FLQ, AMK and ETH perfectly overlapped with the emergence of corresponding known or high-probability resistance-conferring mutations (Figure 2). For STR, however, predictions of drug resistance from the genomic data contradicted the phenotypic drug resistance profiling in one of the serial isolates which had been typed as susceptible. This isolate (SF4) harbored a *gid* nonsense mutation introducing a stop at codon 37. Disruption of *gid* and its 16S RNA-methylase activity is associated with low-level STR resistance [18]. We re-tested the isolates for STR susceptibility and found that isolate SF4 was indeed STR resistant, but grew less efficiently when challenged with STR than subsequent isolates harboring the *rrs* A514C mutation (Figure 3). The last four isolates (SF6 to SF9) were originally typed as EMB resistant, but re-testing did not confirm this. At a lower EMB concentration (2 μg/ml versus 5 μg/ml), however, we found that the last three isolates, harboring non-synonymous *embB* mutations, did indeed grow well, whereas the other isolates did not (Figure 3).

The isolates in the current study never evolved resistance to pyrazinamide (PZA), despite continuous treatment over 3.5 years. Simulations built on an *in vitro* pharmacokinetic-pharmacodynamic model of TB found that the currently recommended PZA dosage (15 to 30 mg/kg body weight/day) would result in sterilizing minimum inhibitory concentration in only 15.1 to 53.3% of patients [22]. It is thus possible that pharmacokinetic factors could explain the inefficacy of PZA in the patient. Clofazimine (CFZ) and CPR were also used together with PZA towards the end of the therapy, and failed to clear the infection despite continued susceptibility to these drug as deemed by DST. By the time these drugs were added to the drug regimen, the patient had developed cavitary TB, a disease state known to be associated with treatment failure [23], presumably due to reduced penetration of drugs in cavities [24]. The development of cavitary TB over the course of infection has most certainly played a role in rendering the infecting strain extremely resilient to antibiotic challenge. Addition of linezolid to the regimen, a drug that has been shown to be effective against cavitary MDR-TB [25,26], finally cleared the infection.

**Figure 4:**
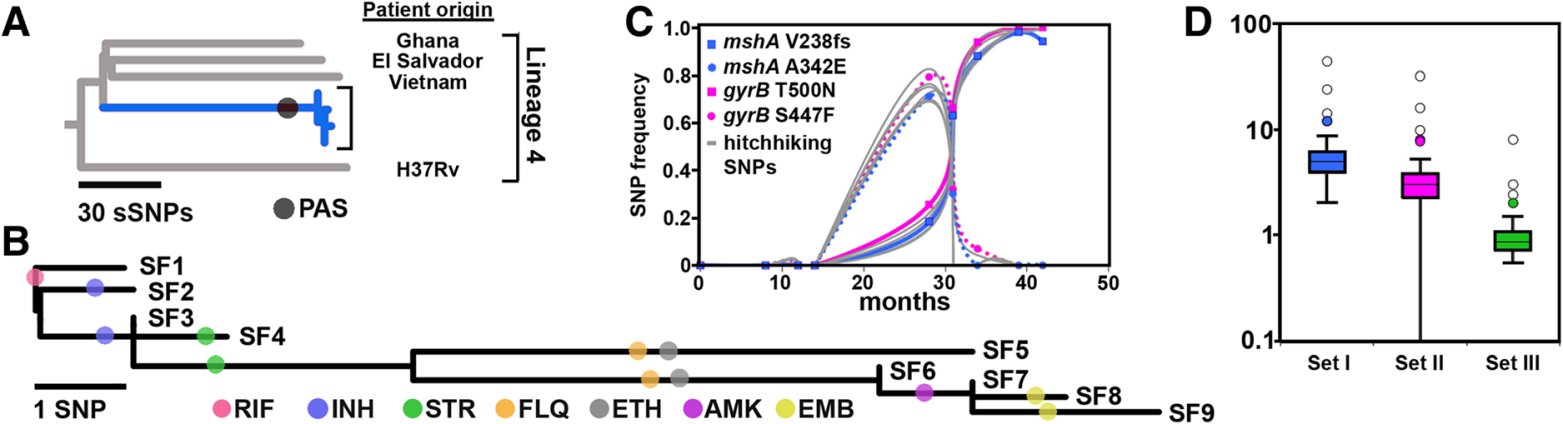
**Intra-patient evolution and mutation rates. (A)** Phylogenetic placement of patient isolates relative to the most closely related isolates from a global *M. tuberculosis* collection (46). A *thyA* P17L mutation possibly conferring para-aminosalicylic acid (PAS) resistance was acquired some time on the branch leading to the patient isolates. sSNP, synonymous SNP. **(B)** Detailed phylogeny of patient isolates with resistance-conferring mutations mapped on the branches. **(C)** Example of hitchhiking SNPs. Frequency of SNPs conferring resistance to FLQ (*gyrB*) and ETH (*mshA*) in sequencing reads over time (months) as well as hitchhiking SNPs (grey shading) with correlation coefficients >0.9 relative to any *gyrB* or *mshA* resistance SNP. **(D)** Box plot of calculated pairwise mutation rates per year between any pair of isolates using three different SNP exclusion criteria: Set I, all SNPs included; Set II, resistance mutations excluded; Set III, resistance mutations and hitchhiking SNPs excluded.

For five out of seven drugs to which the bacillus developed resistance, two independent resistance-conferring mutations emerged, ultimately resulting in fixation of one of the two mutations (Figures 2 and 4B). Only RIF (*rpoB* S450L) and AMK (*rrs* A1401G) resistance were found to result from a single mutation; in both instances the resistance-conferring mutation was among the most commonly observed, and the *rpoB* S450L mutation has previously been shown to carry little or no fitness cost [27].

Extended DST following development of an MDR phenotype revealed that all isolates were resistant to para-aminosalicylic acid (PAS), but as this drug is not part of the standard anti-TB drug regimens nor relevant to the WHO’s definition of MDR and XDR, we refer to the initial isolate (SF1) as susceptible. To place the serial isolates in phylogenetic context and to identify mutations that could be responsible for the pre-existing PAS resistance common to all isolates, we identified single nucleotide variants in a published global collection of *M. tuberculosis* lineage 4 isolates as well as the patient isolates. The analysis resulted in 2,801 synonymous SNPs that were used to construct a maximum likelihood tree (Figure 4A; Figure S1 in Additional file 2; Additional file 3). Next, we investigated all non-synonymous SNPs to identify mutations that could be involved in drug resistance in this patient. Among 94 non-synonymous SNPs unique to the patient isolates (Additional file 3), we found a *thyA* P17L mutation that could explain the observed PAS resistance [28]. Interestingly, the patient isolates also harbored a unique *uvrB* A582V mutation. In *Escherichia coli*, the Uvr(A)BC complex is responsible for excision of a wide variety of DNA lesions. The *uvrB* A582V mutation is localized in a region which in the *E. coli* homologue displays binding affinity towards both UvrA and UvrC [29], and could potentially lead to a reduction in DNA repair capability in the patient isolates.

### Increased fitness in the presence of antibiotics drives clonal expansions

To investigate whether fitness in the presence of antibiotics was driving the expansion of successful clones at the expense of disappearing clones, we assayed growth efficiency of the isolates at a fixed concentration of antibiotics relative to untreated controls for each sample. Challenged with INH and STR, isolates carrying the ultimately fixed INH or STR resistance mutation grew significantly better than isolates harboring the transient mutation, clearly indicating that increased fitness in the presence of antibiotics was driving the expansion of individual clones (Figure 3). For EMB, the picture was similar, but complicated by the fact that EMB treatment was discontinued prior to fixation of any resistance-conferring SNP. The *embB* D1024N mutation was present in almost 5% of the reads in SF7 and was ultimately fixed in SF9. Interestingly, SF8 grew better than SF9 in EMB, indicating that the transient *embB* N644I mutation conferred a higher fitness in the presence of low concentrations of EMB relative to *embB* D1024N. As EMB treatment was discontinued prior to fixation of either mutation, this probably indicates that the *embB* D1024N mutation imparts a lower fitness cost in the absence of drug challenge, and was thus driven to fixation in the population following removal of EMB from the treatment scheme. EMB susceptibility testing has repeatedly been shown to be challenging [30,31]. The *embB* D1024N mutation has previously been described in one isolate characterized as EMB susceptible [32]. However, our results indicate that this mutation confers low-level EMB resistance, illustrating that characterizing resistance mutations conferring borderline resistance remains problematic in a clinical setting. Intriguingly, isolates harboring the same resistance mutations also differed in growth efficiency in the presence of INH and STR, with a clear trend of increasing fitness (Figure 3).

Recent studies have documented the presence of transient genotypes and multiple concomitantly occurring drug-resistance mutations in serial *M. tuberculosis* isolates [11,33,34]. However, our results allowed for an even finer characterization of within-patient microbial dynamics than previous studies thanks to the large number of serial isolates. Taken together, these results point to 'battles among clones' being central to the evolution of drug-resistant *M. tuberculosis*. Clonal expansions seem driven by the increased fitness of mutant strains in the presence of antibiotics relative to the clones they replace.

### Mutation rates are inflated by drug-induced selection

In a recent study of longitudinal *M. tuberculosis* isolates, including all the major lineages, the substitution rate was estimated to 0.5 SNPs per genome per year (95% confidence interval (CI) 0.3 to 0.7) and the divergence was rarely found to be higher than five SNPs per three years [35]. In another study of transmission chains the substitution rate was found to be 0.4 mutations per genome per year [36]. After exclusion of transient mutations in the patient isolates, 4.3 mutations were acquired per year from SF1 to SF9, or 2.3 mutations per year when excluding resistance mutations.

Antibiotic-induced expansion of resistant clones could potentially distort mutation rate estimates as random SNPs in the genetic background of resistant clones sweep to fixation together with the resistance mutation. Our data set allowed us to directly test for this possibility, as a large number of resistance mutations emerged over time and as the frequency of all identified SNPs were known over nine time points. We plotted SNP frequencies over time, from which it became apparent that SNPs not involved in resistance were changing in frequency in concert with the resistance mutation. These SNPs are located in the genetic background of expanding and contracting drug-resistant clones and their frequency changes over time closely mirror those of the resistance SNP due to the absence of genetic recombination in *M. tuberculosis* (Figure 4C). We refer to such SNPs whose allele frequency change is driven solely by linkage to a resistance mutation under natural selection as hitchhiking SNPs.

To investigate the effect of drug-driven selective sweeps on the substitution rate, we calculated pairwise SNP frequency correlation coefficients for all SNPs together with each of the 12 resistance mutations. Hitchhiking SNPs were defined as SNPs not involved in resistance but present at frequencies correlating closely (correlation coefficient >0.9) with any one of the 12 identified resistance mutations over the nine time points. Indels were excluded from these analyses, leaving only true SNPs. Subsequently, pairwise substitution rates were calculated for all possible pairs of isolates, a *de facto* simulation of a situation in which only two random isolates would be available for study, which is the case more often than not in a clinical setting.

Pairwise substitution rates were calculated for the following SNP sets: I) including all SNPs; II) excluding resistance mutations; and III) excluding resistance mutations and hitchhiking SNPs. The calculated mean pairwise substitution rates were found to differ substantially between the three sets (Figure 4D), with a mean mutation rate per genome per year of 7.0 (95% CI 4.5 to 9.4) for set I, 4.3 (95% CI 2.4 to 6.1) for set II and 1.1 (95% CI 0.7 to 1.6) for set III (Figure 4D), demonstrating a massive influence of selection for resistance mutations on substitution rates.

The existence of multiple clonal lineages within a single patient has been well documented in recent reports [11,33,34] and it has also been shown that the diversity of *M. tuberculosis* isolates from a single patient can rival that of different isolates from a transmission chain [33]. In the current work we demonstrate that clinical *M. tuberculosis* isolates descending from a single transmission event can in fact reach a level of divergence within a patient that exceeds the levels normally found between isolate pairs from a transmission chain. Our findings demonstrate that SNPs hitchhiking with resistance mutations can drive a surprisingly large number of SNPs to fixation over a short time span.

By coupling whole genome sequencing and epidemiological information, a recent study found that 96% of paired isolates differed by no more than five SNPs [35]. In the current study, the most extreme SNP divergence over time was found between isolates SF5 and SF6, separated by only three months, yet differing by 11 SNPs. It should be noted that most of the SNPs differentiating SF5 and SF6 were present in both isolates, but the frequency of the SNPs changed dramatically over the three months separating the isolates, reflecting the rapid expansion and collapse of two different clones.

In a typical SNP-calling scheme, mutations at low frequencies are not picked up, and if these were the only isolates available from the patient, it might well have been interpreted as an exogenous reinfection. However, based on the analysis of nine serial isolates, we can safely conclude that the dominant clones in isolates SF5 and SF6 shared a common ancestor that existed in the very patient they were isolated from. After removal of resistance mutations as well as hitchhiking mutations, the total number of mutations separating the nine isolates decreased from 24 to 4, a clear indication that antibiotics-induced selection was the main driver of the observed diversification over time. The high number of hitchhiking SNPs may suggest that the rapid emergence of resistance is facilitated by large populations within the host harboring considerable standing variation (many variants at very low allele frequency), which might not be picked up even when sequencing at >100× coverage.

### Genes involved in mycolic acid synthesis and drug efflux are differentially expressed among serial isolates

**Figure 5:**
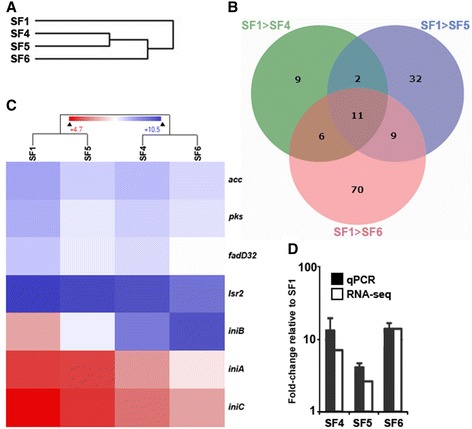
**Differential gene expression in serial isolates. (A)** Hierarchical clustering of total gene expression. **(B)** Venn diagram of differentially expressed genes in SF4, SF5 and SF6 relative to SF1. **(C)** Hierarchical clustering of interesting genes and operons (high and low expression indicated by blue and red coloring, respectively). **(D)** Fold change of *iniB* expression relative to SF1. qPCR, quantitative PCR.

We selected four isolates, SF1, SF4, SF5 and SF6, for RNA sequencing, as these represented the initial isolate, as well as the isolates in which the main bulk of resistance development took place. Hierarchical clustering of total gene expression levels revealed that transcription patterns roughly reflected the phylogeny of the isolates (Figures 4B and 5A). Analyses of differential expression were performed for SF4, SF5 and SF6 independently with SF1 as the reference.

A total of 139 genes were found to be differentially regulated between at least one of the later isolates (SF4, SF5 and SF6) and SF1 (Figure 5B; Additional file 4). None of the mutations emerging over time in the clinical isolates were located in genes or promoter regions of genes for which significant differential transcription was observed, demonstrating that differential expression was not directly attributable to mutations in corresponding gene regions (Additional files 1 and 4).

Clusters of orthologous groups (COG) analyses were carried out for up- and down-regulated genes (Table S1 in Additional file 2). In all the later isolates, significantly down-regulated genes relative to SF1 were enriched for the COG category 'Secondary metabolites biosynthesis, transport and catabolism' (*P*-values of 0.057, 0.019 and 0.016, respectively, for SF4, SF5 and SF6 relative to SF1). Among the down-regulated genes were *pks13* and *fadD32*, members of the *pks13-fadD32-accD* operon, encoding enzymes that are responsible for the final steps of mycolic acid synthesis [37]. Down-regulation of this operon could possibly be involved in adaptation to INH and ETH treatment, both of which target steps in mycolic acid synthesis.

Transcription of *iniA*, a member of the *iniBAC* operon, involved in drug efflux and previously shown to confer increased resistance to both INH and EMB [38], was significantly up-regulated in SF6 relative to SF1. To investigate transcription levels of the *iniBAC* operon in more detail, we assayed *iniB* expression by quantitative PCR, which confirmed upregulation of the gene in SF4 to SF6 relative to SF1 (Figure 5). Concomitant with *iniBAC* up-regulation, we observed a slight down-regulation of *lsr2*, a known negative regulator of *iniBAC* expression [39].

We observed an increase in growth efficiency over time that was independent of the specific resistance-conferring mutations (Figure 3). This trend was most striking when the isolates were grown in the presence of INH and the most parsimonious explanation would be that transcriptional reprogramming serves as an additional layer for boosting fitness in the presence of drugs. We did indeed observe patterns of differential gene expression that could be involved in a systemic adaptation to challenge with multiple compounds, namely an upregulation of *iniBAC* expression combined with the down-regulation of *pks13* and *fadD32*, encoding proteins responsible for the final steps of mycolic acid synthesis.

Down-regulation of mycolic acid synthesis could potentially alleviate detrimental effects of accumulated intermediaries upon inhibition in InhA. The isolates in which *pks13* and *fadD32* were down-regulated harbored a *katG* P7 frameshift mutation resulting in a premature stop codon. However, *katG* has an alternative start codon in position +76 in the same frame as the annotated start codon. Inspection of RNA-seq reads aligned to the H37Rv reference genome revealed that *katG* was transcribed in full length also in the isolates harboring the frameshift mutation, despite a rather steep decline in transcription levels downstream of the new stop codon (Figure S2 in Additional file 2). It is thus plausible that some KatG activity remained to activate INH. WhiB7 was previously found to orchestrate a transcriptional response to diverse classes of antibiotics, conferring increased drug tolerance [40]. Our results support a role of transcriptional responses in modulating drug susceptibility. As RNA was isolated from bacteria grown in the absence of antibiotics, this finding indicates that stable transcriptional changes had taken place.

Another possible explanation for the increased fitness in the presence of INH and STR over time could be a cumulative effect of multiple mutations. An *ahpC* N126D mutation was common to isolates SF5 to SF9 and might have increased the resistance to INH conferred by the *katG* frameshift mutation. Promoter mutations resulting in *ahpC* overexpression have been linked to compensation for loss of *katG*-associated catalase activity [41]. However, the *ahpC* N126D mutation is located in the body of the gene, and as gain-of-function mutations are rare, it seems unlikely that this mutation played a significant role in INH resistance or fitness compensation. It is also possible that the *rrs* A1401G mutation conferring KAN/AMK resistance could yield increased resistance to STR in combination with the *rrs* A514C mutation. However, this scenario also seems relatively unlikely as such an effect has never been reported despite these being common and well-studied mutations. We did not identify other obvious candidate mutations that could explain this trend. Recent *in vitro* studies have shown that the efflux inhibitor verapamil potentiates the antitubercular effect of bedaquiline and CFZ and that efflux contributes to the emergence of MDR-TB [42,43]. Our findings suggest that drug efflux could be an important mechanism effectuating drug resistance also within patients.

## Conclusions

The availability of nine serial isolates combined with deep sequencing gave us unprecedented insights into the dynamics of the emergence of drug resistance in *M. tuberculosis*. A surprisingly large number of SNPs were found to differentiate serial *M. tuberculosis* isolates evolving resistance to multiple drugs within a single patient. Most of the mutations were either resistance SNPs or hitchhiking SNPs in the genetic background of resistance mutations. This finding has major implications for the reconstruction of transmission chains between patients under treatment. In particular, the large number of SNPs we detected between isolates sampled only a few months apart suggests that using a simple threshold of a maximal number of mutations between pairs of isolates to rule out direct transmission may often be inaccurate.

Mutations underlying antibiotic resistance evolving over the course of infection were easily identifiable. However, over the course of infection, the *M. tuberculosis* isolates exhibited increased fitness in the presence of antibiotics that was independent of any obvious resistance-associated mutations. We did, however, identify patterns of differential gene expression that could explain the observed systemic adaptation to challenge by multiple compounds, including an up-regulation of the efflux-associated *iniBAC* operon over time. This observation suggests that drug efflux could be an important mechanism effectuating drug resistance within patients. Drugs targeting either drug efflux or transcriptional regulators that coordinate the response of *M. tuberculosis* to antimicrobial compounds could thus prove valuable in combination with conventional anti-TB drugs.

## Materials and methods

### Patient clinical information and phenotypic resistance

An immigrant from Eastern Europe was diagnosed with non-cavitary pulmonary TB at an outpatient clinic in Norway. *M. tuberculosis* isolates from sputum were sensitive to the conventional TB drugs. The patient was treated with a standard anti-tuberculous regimen consisting of INH, RIF and PZA. It is not known if the patient received DOT at the time. After eight months live bacilli were still isolated from the sputum, a cavity in the right upper lobe had developed and by this time the patient had developed MDR-TB resistant to INH and RIF. The patient was then transferred to Oslo University Hospital where the patient received DOT. The treatment was changed to STR and EMB in addition to PZA. Ofloxacin (OFX) and ETH were added to the scheme shortly after. During the following year tubercle bacilli were not isolated from the patient. However, two years after initiation of the first therapy regimen, *M. tuberculosis* was again isolated and had now acquired resistance to STR, OFX, ciprofloxacin and ETH. STR, ETH and OFX treatment was thus discontinued whereas PZN and EMB were continued and AMK and CFZ were added to the scheme. Three months later the bacterium had developed resistance to EMB and AMK, and thus fulfilled the diagnosis of XDR-TB. These drugs were discontinued and replaced with CPR, amoxicillin/clavulanate, levofloxacin, gemfibrozil and interferon gamma-1b inhalations. The three latter experimental drugs were discontinued after a short period, leaving PZN, CFZ and CPR as the regimen. The cavity in the right lung persisted and upper lung lobectomy was performed on the infected lung but failed to clear the infection. Finally linezolid was added to the therapy and the sputum became culture-negative three weeks later. A full pneumectomy was performed shortly after due to massive chronic tissue damage.

### Isolates and ethics approval

Ethics approval for this study was obtained from the Norwegian Regional Ethics committee (reference number 2014/191). Primary sputum cultures were collected at Oslo University Hospital Ullevål and final DST and molecular epidemiological typing performed at the Norwegian Institute of Public Health (NIPH). All isolates were typed by IS*6110* RFLP and 24-locus MIRU-VNTR [44], both methods yielding nine identical profiles.

### Drug susceptibility testing and growth experiments

DST was performed on the BD BACTEC 460 (BD Diagnostics) radiometric system with the following critical concentrations: 6 μg/ml STR, 0.2 μg/ml INH, 2 μg/ml RIF, 7.5 μg/ml EMB, 2.0 μg/ml OFX, 2.0 μg/ml ciprofloxacin, 5 μg/ml ETH, 100 μg/ml PZA, 4.0 μg/ml AMK, 4.0 μg/ml KAN, 4 μg/ml PAS and 10 μg/ml CPR. Re-testing of STR and EMB susceptibility was carried out on the BD MGIT™960 fluorometric system with standard concentrations: 1 μg/ml and 4 μg/ml for STR and 5 μg/ml for EMB. To assay fitness under antibiotic challenge, experiments were performed in triplicates with the following treatments: no drug (control experiments); 1 μg/ml STR, 0.4 μg/ml INH or 2 μg/ml EMB. Colonies were picked from solid Löwenstein-Jensen (LJ) medium, adjusted to McFarland turbidity 0.5 and inoculated in MGIT tubes following standard procedures. To quantify growth efficiency under drug challenge, growth unit (GU) read-outs of the corresponding drug-treated sample at the time when the growth control reached 400 GU was used.

### Genome sequencing and analysis

Genomic DNA was isolated from a full loop of colonies growing on LJ medium using a Bacterial DNA Kit (Omega Bio-tek, Norcross, Georgia, USA) following the manufacturer’s instructions with the following modifications. A 20 minute incubation at 94°C to inactivate the bacteria was added after resuspension of bacteria in TE buffer. Lysozyme incubation was performed for 60 minutes at 37°C followed by bead beating 3 × 6,800 rpm for 30 seconds in Precellys Tough micro-organism lysing tubes in a Precellys 24 biological grinder (Bertin Technologies, Ampère, Montigny-le-Bretonneux, France) Genomic DNA (500 ng) was used to generate sequencing libraries. DNA was fragmented with NEBNext dsDNA fragmentase (NEB, Ipswich, Massachusetts, USA) for 45 minutes according to the supplied protocol. Fragmented DNA was purified with Agencourt AMPure beads and Illumina sequencing libraries generated with the High Throughput Library Preparation Kit (KAPA Biosystems, Wilmington, Massachusetts, USA) following the manufacturer’s protocol. Individual libraries were indexed with NEXTflex barcodes (Bioo [SIC] Scientific, Austin, Texas, USA) and sequenced on both the Illumina HiSeq and MiSeq platforms with 50 bp single end and 150 bp paired-end run modes, respectively. Fastq reads were aligned to the H37Rv genome with SeqMan NGen (DNASTAR), resulting in a median coverage of 210× (189× to 246×).

We first called SNPs in SeqMan Pro (DNASTAR) that were present at a minimum depth of 50 and at a minimum frequency of 25% reads in any one sample. Second, we re-called SNPs at these positions in all samples, allowing for a SNP frequency as low as 4%. Fixed mutations were defined as being present in ≥85% of the reads in the final isolate (SF9). Only SNPs found at a frequency of >70% in at least one sample were used to construct a neighbor-joining phylogeny of SF isolates. In any isolate, variants at these positions were called as SNPs if the frequency was above 50%. For comparative genomic analyses of SF isolates with a global collection of lineage 4 isolates (Additional file 3), paired-end reads were downloaded from the NCBI Short Read Archive (ERP001731). Reads were aligned as above. SNPs were called using a haploid Bayesian approach in SeqMan NGen with the following thresholds: SNP% 95, depth 20, Q60. Repetitive regions including PE/PPE genes and SNPs occurring at a distance of 10 bp or less relative to these or each other were excluded from all analyses. A PhyML phylogeny was constructed using SeaView [45].

Selected SNPs were verified by mutation-specific PCR using primers specifically amplifying either the wild-type or mutant allele (Table S2 in Additional file 2). PCR was performed on a Roche LightCycler® real-time PCR machine using KAPA SYBR FAST master mix (KAPA) and 20 ng template DNA in 20 μl PCR reactions with the following cycling parameters: preincubation 95°C 1 minute; cycling 3 s at 95°C, 30 s at 70°C (Roche, Basel, Switzerland). For each primer pair, thermal cycling was terminated when the earliest amplification reaction was in late logarithmic phase, and amplicons were visualized on ethidium bromide-stained agarose gels.

### RNA sequencing and analysis of gene expression

For each of the three replicates per isolate, a full loop of bacterial colonies picked from solid LJ medium was resuspended in 1 ml Tri reagent (Sigma-Aldrich, St Louis, Missouri, USA) in Precellys tubes. The bacteria were bead-beaten twice at 6,800 rpm for 30 s and cooled on ice between and after beating steps. After beating, 0.2 ml of chloroform was added directly to the Precellys tubes, vortexed and centrifuged at 12,000 × g for 15 minutes at 4°C. RNA was precipitated from the aqueous phase with 0.5 ml isopropyl alcohol, incubated at room temperature for 10 minutes and centrifuged at 12,000 × g for 10 minutes at 4°C. The RNA precipitate was washed once with 75% ethanol, air dried and dissolved in 30 μl nuclease-free water, followed by DNAse treatment of the RNA with the DNA-free RNA kit (Zymo Research, Irvine, California, USA). For each replicate, rRNA was partially depleted from 3 μg RNA using the Ribominus Transcriptome Isolation Kit (Invitrogen, Carlsbad, California, USA). Enriched mRNA was purified on Zymo RNA Clean & Concentrator columns (Zymo) and RNA-sequencing libraries constructed with NEBNext® Ultra Directional RNA Library Prep Kit (NEB) and indexed with NEBNext multiplex oligos for Illumina (NEB). Libraries were sequenced on the Illumina HiSeq 2000 platform with 50 bp single end run mode.

Reads were aligned to the *M. tuberculosis* H37rv reference genome in SeqMan NGen. BAM assemblies were imported into QSeq (DNASTAR). Experiments were normalized by assigning reads per kilobase template per million mapped reads (RPKM) and scaled to have a common mean. Significant differential expression was defined as *P* <0.05 after adjustment for multiple testing using the Benjamini-Hochberg false discovery correction. Complementary DNA was synthesized from RNA using a ProtoScript II First Strand cDNA Synthesis Kit with random primers (NEB) according to the manufacturer's instructions. cDNA diluted 1:5 was used as template in 20 μl reactions. The primers used for amplification of *iniB* and *rpoB* are listed in Table S1 in Additional file 2. Standard curve templates were generated by preparation of 10-fold dilution series of pooled cDNA from all samples (undiluted to 10^-4^ dilution). Expression levels of *iniB* were normalized to *rpoB* expression across samples. COG analyses were performed using the TB database [46].

### Data access

Sequence reads were deposited at the European Nucleotide Archive with the following accession numbers: [EMBL:PRJEB5899] (genome sequences) and [EMBL:PRJEB5865] (RNA sequences).

## Additional files

Additional file 1:
**SNPs differentiating the nine clinical TB isolates.**
Additional file 2:
**Supplementary Tables and Figures.**
Additional file 3:
**Synonymous SNPs identified in the nine serial TB isolates and isolates from a global collection of TB isolates and non-synonymous SNPs unique to the serial isolates.**
Additional file 4:
**Differentially regulated genes identified by RNA-seq.**

## Abbreviations

AMK: amikacin
bp: base pair
CFZ: clofazimine
CI: confidence interval
COG: clusters of orthologous groups
CPR: capreomycin
DOT: directly observed treatment
DST: drug susceptibility testing
EMB: ethambutol
ETH: ethionamide
FLQ: fluoroquinolone
GU: growth unit
INH: isoniazid
KAN: kanamycin
LJ: Löwenstein-Jensen
MDR-TB: multidrug-resistant tuberculosis
OFX: ofloxacin
PAS: para-aminosalicylic acid
PCR: polymerase chain reaction
PZA: pyrazinamide
RIF: rifampicin
SNP: single-nucleotide polymorphism
STR: streptomycin
TB: tuberculosis
XDR-TB: drug-resistant tuberculosis
