# Supplementary material for: Evolution of extensively drug-resistant Mycobacterium tuberculosis from a susceptible ancestor in a single patient
Source: Genome Biol. 2014 Nov 7;15(11):490. doi: 10.1186/s13059-014-0490-3 (PMC4223161; doi:10.1186/s13059-014-0490-3)
Supplement: Additional file 2: — Supplementary Tables and Figures. [file 13059_2014_490_MOESM2_ESM.pdf]

Supplementary tables and figures.

Table S1. Cluster of orthologous groups of significantly up- and downregulated genes in isolates SF4-SF6 relative to SF1. Significantly enriched categories are highlighted in bold.

	COG term	Specified genes (n)	Genome (n)	specified genes (%)	Genome (%)	p value	corrected p value
**1>4 down**	Secondary metabolites biosynthesis transport and catabolism	2	240	50	6	0.019	0.057
	Signal transduction mechanisms	1	103	25	2.58	0.095	0.286
	General function prediction only	1	441	25	11.03	0.311	0.933
**1>4 up**	Cell motility	1	11	16.67	0.28	0.016	0.081
	Energy production and conversion	2	218	33.33	5.45	0.036	0.178
	Inorganic ion transport and metabolism	1	125	16.67	3.13	0.16	0.801
**1>5 down**	**Secondary metabolites biosynthesis transport and catabolism**	4	240	36.36	6	0.003	**0.019**
	Signal transduction mechanisms	2	103	18.18	2.58	0.029	0.201
	Nucleotide transport and metabolism	1	64	9.09	1.6	0.15	1.051
**1>5 up**	Carbohydrate transport and metabolism	2	114	10.53	2.85	0.085	0.934
	Translation ribosomal structure and biogenesis	2	121	10.53	3.03	0.093	1.021
	Inorganic ion transport and metabolism	2	125	10.53	3.13	0.097	1.071
**1>6 down**	**Secondary metabolites biosynthesis transport and catabolism**	6	240	26.09	6	0.002	**0.016**
	Signal transduction mechanisms	3	103	13.04	2.58	0.018	0.177
	Lipid transport and metabolism	3	263	13.04	6.58	0.129	1.294
**1>6 up**	Cell wall/membrane/envelope biogenesis	4	108	10	2.7	0.018	0.231
	General function prediction only	8	441	20	11.03	0.04	0.516
	Secondary metabolites biosynthesis transport and catabolism	5	240	12.5	6	0.058	0.76

Table S2. Oligonucleotides listed by application

Primer	Sequence	Application
gyrB_F	GGCACGAGAGTTGGTGCGGCG	
gyrB_SNPR1	CGGACTTGCGCGGATCCGTGA	
gyrB_SNPR2	CCGTGATGATCGCCTGAACTTCGT	
gyrB_WTR1	CGGACTTGCGCGGATCCGTGG	
gyrB_WTR2	CCGTGATGATCGCCTGAACTTCGG	
embB_F1	GTCGCGCAACCGGATGGCGTTC	SNP verification
embB_SNPR1	AGCTGGAGACATACCACCAGCCGA	
embB_WTR1	AGCTGGAGACATACCACCAGCCGT	
embB_F2	CTGCTGGACTGGGCGGTCGGT	
embB_SNPR2	GCCGTCTTCCCACGTGTCGGTGTT	
embB_WTR2	GCCGTCTTCCCACGTGTCGGTGTC	
iniBF	CAAATCTCATCGGTGGCGGCCAATG	
iniBR	CGCAATCCGCTCATGGGGTCG	qPCR
rpoBF	GCCTGCGTACGGTCGGCGA	
rpoBR	GCCTCCACGTCCTGGGTGGTC	

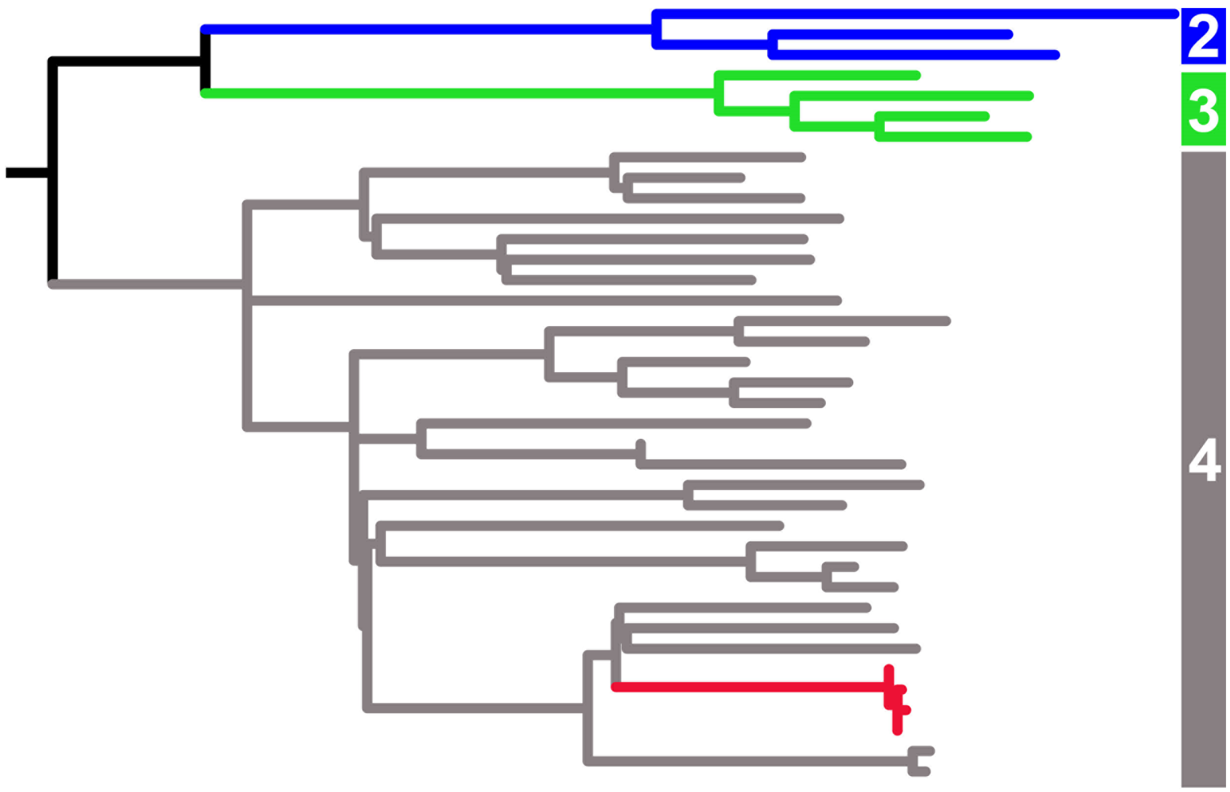

Figure S1. Phylogenetic placement of patient isolates. Maximum likelihood tree based on 2801 synonymous SNPs. Numbers refer to the modern *M. tuberculosis* lineages 2,3 and 4.

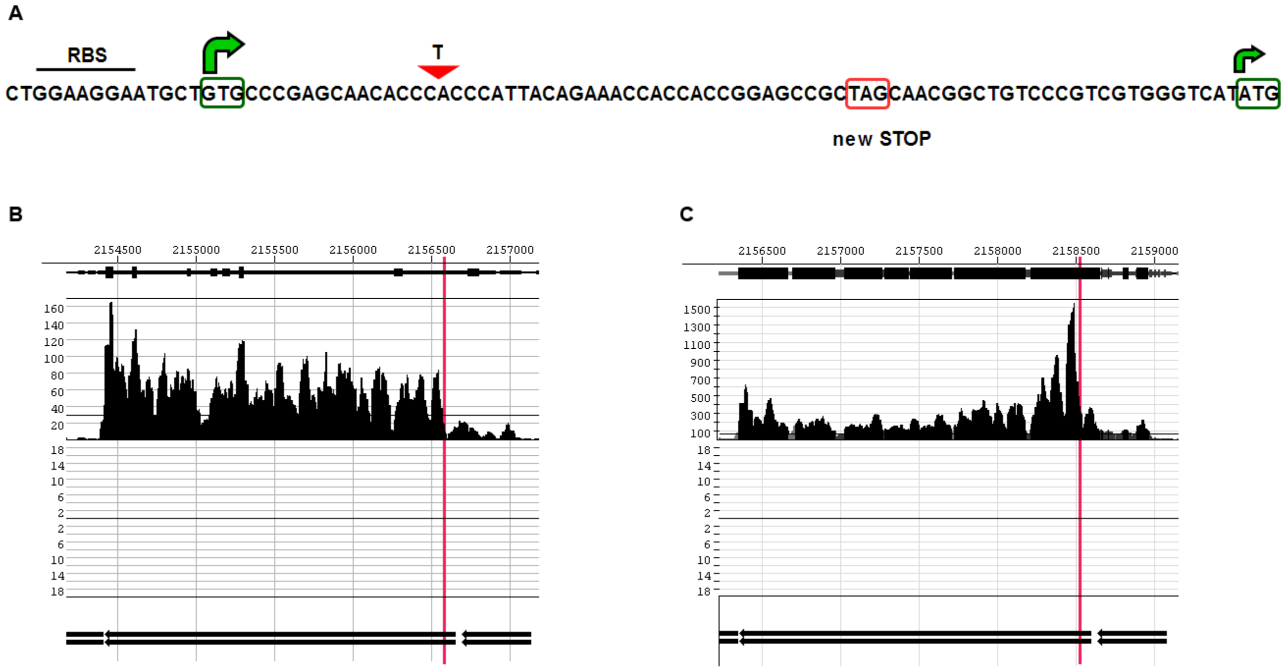

Figure S2. Expression of *katG*. (A) Schematic overview of *katG* features. Ribosomal binding site (RBS) and start codon (GTG) are indicated. The insertion identified in isolates SF2-SF9 is indicated by a red triangle. The insertion results in a premature STOP codon which is followed by an alternative ATG start codon. (B) RNA-seq reads from isolate SF1 aligned to *katG*. (C) RNA-seq reads from isolates SF4-SF6 aligned to *katG*.
